# Dahlia Virome Analysis Reveals Multiple Novel Viruses in Mixed Infections

**DOI:** 10.3390/v18090947

**Published:** 2026-08-29

**Authors:** Peter Abrahamian, Crosley Kudla-Williams, Samuel Grinstead, Katie Posis, Nicholas Winarto, Tongyan Tian

**Affiliations:** 1 USDA Agriculture Research Service (USDA-ARS), National Germplasm Resources Laboratory, Beltsville, MD 20705, USA; 2 USDA Agriculture Research Service (USDA-ARS), Molecular Plant Pathology Laboratory, Beltsville, MD 20705, USA; 3 Plant Pest Diagnostics Center, California Department of Food and Agriculture, Sacramento, CA 95832, USA

**Keywords:** ornamentals, emerging viruses, ophiovirus, potyvirus, yuevirus, partitivirus, high throughput sequencing, virions, electron microscopy

## Abstract

Dahlia is an economically important ornamental crop in the United States and worldwide. Dahlia is primarily propagated vegetatively through bulbs, which can result in vertical transmission of and constant accumulation of viruses. In this study, mosaic and yellowing symptoms were observed in Dahlia plants in a residential garden in California. Dahlia samples were collected and sequenced using high-throughput sequencing (HTS). HTS analysis was used to explore the Dahlia virome in the symptomatic plants, which revealed the presence of several contigs with homology to known Dahlia-infecting viruses, such as Dahlia common mosaic virus (DCMV) and tobacco streak virus (TSV), and Dahlia latent viroid. However, two contigs showed that they belong to the genera *Potyvirus* and *Miraophiovirus* with low to medium homology to previously known *Potyvirus* and *Miraophiovirus* species. Sequence identity showed that these two contigs represent two novel viruses tentatively named Dahlia potyvirus 1 (DaPoV1) and Dahlia miraophiovirus 1 (DaMV1). In addition, two other contigs showed low homology to a partitivirus and yue-like virus, indicating novelty. Mechanical inoculation of infected Dahlia tissue did not result in successful transmission onto indicator hosts. Electron microscopy of symptomatic plants showed typical potyviral-like flexuous virions. Phylogenetic analysis confirmed the placement of DaPoV1 and DaMV1 in their respective genera. This study represents a significant expansion of the known Dahlia virome and uncovers the complexity of viral infections where DCMV and TSV have been historically the primary focus in Dahlia. Widespread surveys should reveal the distribution of these viruses at commercial production sites.

## 1. Introduction

The genus *Dahlia* (family *Asteraceae*) represents one of the most genetically diverse and horticulturally significant groups of ornamental plants [1]. Dahlias are native to Central America and have been bred over many years into thousands of cultivars, ranging from the petite types to massive varieties [1,2]. In the U.S., Dahlia is a popular cut flower, and many different varieties are grown for local production [3]. Dahlias are primarily propagated vegetatively through tubers, which makes them highly susceptible to the accumulation and transmission of viral pathogens [4]. Once a virus enters a Dahlia plant line, it gets passed down through every subsequent generation of tubers. Dahlias are also propagated through seeds, which can also carry persistent viruses [4,5].

Historically, viral diseases in Dahlia have been dominated by Dahlia mosaic virus (DMV) and Dahlia common mosaic virus (DCMV), both of which belong to the Caulimovirus genus [6]. Earlier reports indicated both DMV and DCMV as separate species; however, recent studies have shown both to be variants of the same species [7,8]. DCMV exists both as an episomal virus (causing disease) and as endogenous viral elements (EVEs) integrated into the Dahlia genome, which can complicate molecular diagnostics [7,9]. For the purposes of this manuscript, DCMV will be used as the caulimovirus causing disease in Dahlia and DMV-D10 as an EVE in accordance with the nomenclature suggested by Geering et al. [7]. Another widespread virus is tobacco streak virus (TSV) in the genus *Ilarvirus* and cucumber mosaic virus to a lesser extent in *Cucumovirus*, both in the family *Bromoviridae.* TSV often appears in mixed infections with DCMV, leading to necrotic streaks or chlorosis [10,11]. Several orthotospoviruses, such as tomato spotted wilt virus, which is somewhat widespread, tomato yellow ring orthotospovirus, and Impatiens necrotic spot virus, cause ring spots, yellowing, and systemic wilting [11]. Viroids such as Dahlia latent viroid, Chrysanthemum stunt viroid, tomato chlorotic dwarf virus, and potato spindle tuber viroid have also been reported [11,12]. For a comprehensive list of viruses in Dahlia, see Sastry et al. [11]. The primary diagnostic challenge is that many of these viruses occur as mixed infections and some share similar symptoms. Therefore, a plant exhibiting mosaic or yellowing symptoms may be harboring a mixture of two or more different viruses.

For decades, virus detection in Dahlia relied on targeted methods like ELISA or RT-PCR. These methods work well but rely on prior knowledge of the target pathogen, which is a major weakness [13,14,15,16]. High-throughput sequencing (HTS) has revolutionized this field by providing an untargeted approach to the plant virome [16]. Furthermore, HTS provides a quantitative and qualitative map of every virus present in a single sample, allowing researchers to see how known viruses like DCMV might interact in a background virome [17,18]. By providing full-length or near-full-length genome sequences, HTS allows for precise phylogenetic placement, helping distinguish between different strains of a virus [18,19]. The sensitivity of HTS can uncover viruses that are present in concentrations too low for traditional assays but may still contribute to long-term cultivar impact [14,15]. In the 21st century, HTS is the definitive tool for moving beyond simple positive or negative testing toward a comprehensive understanding of the viral ecology within the host [17,20]. The use of HTS has resulted in a significant expansion in the viromes of several crops and viral taxonomy, with new families, genera, and species reported annually [21].

Several viruses like DCMV, TSV, and TSWV, and others, are well-documented to cause yellowing or mosaic symptoms [11]. Despite rigorous testing for these viruses, plants frequently exhibit unexplained mosaic and mottling symptoms. Traditional diagnostics often miss novel or low-titer viruses that may contribute to viral complexes. In this study, several Dahlia plants from a local California nursery exhibited yellowing and mosaic symptoms and were obtained for laboratory diagnostics. Initial testing using PCR-based protocols revealed the presence of DCMV and a low homologous sequence to tobacco etch virus. Therefore, the objectives of this study were to determine the etiology of yellowing and mosaic symptoms in Dahlia nursery plants using HTS, to validate the biological relevance of newly discovered viral species, and to report the Dahlia virome.

## 2. Materials and Methods

### 2.1. Plant Material

In 2022, symptomatic leaf samples from Dahlia cv. Marilyn (designated sample 22-1) and Dahlia cv. Crichton Honey (designated sample 22-2) from a residential site were received at CDFA for testing. In 2024, two Dahlia cv. Marilyn plants were re-sampled from a residential site, and tubers were shipped to NGRL for grow-out assays and further testing. A total of six tubers from the two different plants (designated 24-1 and 24-2) were grown out in 6″ pots in a Metro-Mix 852 (Sun Gro Horticulture, Agawan, MA, USA) potting mix. Tubers were germinated and maintained at a temperature of 21 to 23 °C in an insect-proof glasshouse and fertilized periodically. All six plants that germinated from tuber samples were further used for leaf tissue collection for biological assays and molecular analysis. The plants were maintained in the greenhouse year-round to observe flowering. Dahlia leaf samples of 6 different varieties, Alauna Millipiques, Normandy Deegee, Lover Boy, Gypsy Night, Ferncliff Dolly, Ginger Crush, and tubers of variety BQ Curt, were also collected and tested from a nursery.

### 2.2. Leaf Dip Transmission Electron Microscopy

Initially, symptomatic samples were used to prepare leaf dips for rapid screening of virus particles using transmission electron microscopy (TEM) in accordance with Mayhew and Flock [22]. In addition, 4 g of leaf tissue were partially purified using procedures according to Lane 1992 in minor modification as previously described [23]. Purified virions were negatively stained using 1% phosphotungstic acid pH 7.0 with 100 µg bacitracin per mL and analyzed using a Hitachi H7500 (Hitachi, Ltd., Tokyo, Japan) transmission electron microscope (TEM). Images were taken using a BioSprint 12M CCD camera (AMT Corp, Danvers, MA, USA), and virions were measured using the camera software.

### 2.3. Preliminary Virus Detection

Marilyn and Crichton Honey samples were tested using PCR for DCMV and potyviruses. Briefly, nucleic acid was extracted from both samples using the MagMAX-96 viral RNA isolation kit (Ambion, Austin, TX, USA) according to the procedures described by Diaz-Lara et al. [24]. An RT-PCR assay using generic potyvirus primers CIFor/CIRev and HPFo/HPRev targeting the *CI* and *HC-Pro*, respectively, by Ha et al. [25] was performed on the diseased samples. To detect the novel potyvirus, DaPoV1, specific primer pairs (Dah-HP-cdfaF1/R1) were designed that amplify a 531 bp fragment from the *HC-Pro* gene. Also, DCMV was attempted in RT-PCR using previously designed primer pairs [5,10]. PCR amplicons were purified and directly sequenced at Azenta Life Sciences (South San Francisco, CA, USA).

### 2.4. HTS and Bioinformatics

Total RNA was extracted from leaf tissues of the Marilyn sample from 2022 and from two germinated mother tuber samples #1 and #2 using the Plant RNeasy extraction kit (Qiagen, Hilden, Germany) according to the manufacturer’s instructions. RNA quality was evaluated on a TapeStation (Agilent Technologies, Santa Clara, CA, USA). RNA libraries were prepared using the TruSeq Stranded Total RNA with Ribo-Zero Plant (Illumina Inc., San Diego, CA, USA) and sequenced using a 1 × 100-bp single-end read kit on a NextSeq 2000 (Illumina Inc.) benchtop sequencer. Raw reads were trimmed for quality and adapters using Trimmomatic 0.39 [26] and assembled de novo using SPAdes 3.15.5 [27] with the rnaviralspades preset. Assembled contigs were identified by performing blastx (v2.13) searches against a custom database generated from RefSeq virus proteins and the *Arabidopsis* genome. Contigs matching *Arabidopsis thaliana* and *A. lyrata* genomes were used for plant contig subtraction and removed from downstream analysis; the remaining contigs were used for blastx against the full NCBI nr database. Sequence homology was determined based on the highest bit score, lowest E-value, and percent identity. Trimmed HTS reads were used to map back to the viral contigs with bbmap 39.01 [28] with minid = 0.76.

### 2.5. RT-PCR Validation of HTS Data

To validate the contigs using HTS, primers were either designed or obtained from the literature. To detect the novel miraophiovirus contigs, three primer pairs that target each RNA segment were designed. To validate the presence of the novel potyvirus, DaPoV1, specific primer pairs (Dah-HP-cdfaF1/R1) were used. To detect DCMV, TSV, and DLVD, primers were designed based on the contig sequences obtained in the datasets or from the literature. To detect the partitivirus and yue-like viruses, two sets of primers were designed in this study to amplify fragments of the respective contigs. All amplicons were directly sequenced using Sanger sequencing at MCLAB (San Francisco, CA, USA). Primers designed in this study for virus detection and RACE PCR are listed in Table 1 and Supplementary Table S1, respectively.

### 2.6. RACE-PCR

To determine the 5′ and 3′ terminal sequences of the novel potyvirus, miraophiovirus, yuevirus, and partitivirus, Rapid Amplification of cDNA Ends (RACE) PCR was performed. The RACE 5′ sequences were determined by using the Template Switching Enzyme (New England Biolabs, Ipswich, MA, USA) and a hybrid RNA: DNA template switching oligo according to the manufacturer’s instructions. A nested PCR was performed using a TSO-specific primer and a downstream virus-specific primer for the potyvirus and miraophiovirus contigs. To determine the RACE 3′ for the potyvirus, the native poly(A) tail was utilized by using an oligodT anchor and an upstream gene-specific primer. To determine the RACE 3′ sequence for the miraophiovirus, partitivirus, and yuevirus segments, total RNA was used as a template for poly (A) tailing using the *E. coli* poly (A) polymerase (NEB). Subsequently, the a-tailed RNA was used as a template for cDNA synthesis using an oligodT anchor primer and upstream gene-specific primer. The resulting fragments were cloned into the pCR4 vector and sequenced at MCLAB to obtain the full-length genome coverage.

### 2.7. Transmission Experiments

Mechanical inoculation was performed to assess the host range and transmissibility of the novel potyvirus. Sap from symptomatic dahlia leaves was extracted in 0.1 M potassium phosphate (K_2_HPO_4_) buffer, pH 7. Briefly, 20 to 30 mg of symptomatic leaf tissue from each of the two unique germinated mother plant tubers above were collected and ground in 2 mL phosphate buffer and kept on ice until inoculation. Mother plants used for inoculation experiments were previously tested using HTS, described above. Each virus-infected sap sample was inoculated on six indicator plants, for a total of 12 plants. The two youngest, fully expanded leaves on each indicator plant were used for inoculation. Carborundum was dusted on the leaves as an abrasive, and 50 µL of the inoculum was pipetted onto each leaf. The inoculum was gently rubbed onto each leaf by hand. A mock-inoculated control was included for each set of indicators by using 50 µL per leaf of phosphate buffer. A total of 11 plant species were grown in a growth chamber from seed: *Capsicum annum* cv. California Wonder, *Chenopodium quinoa*, *Cucumis sativus*, *Datura stramonium*, *Nicotiana benthamiana*, *Nicotiana edwardsonii*, *Nicotiana glutinosa*, *Nicotiana tabacum*, *Physalis pubescens*, *Solanum melongena* cv. Nigral (F1), *Solanum lycopersicum* cv. Glacier Tomato. Symptom development was assessed at 7, 14, and 28 days post-inoculation (dpi). The indicator plants were sampled at 14 and 28 dpi by collecting two to three 9 mm leaf disks from the youngest fully expanded leaf on each plant. Leaf disks were stored at 4 °C until being used for RNA extraction and RT-PCR testing. Experiments were repeated twice.

### 2.8. Phylogenetic Analysis

Phylogenetic trees were constructed to determine the evolutionary relationship of novel viruses. Amino acid sequences of the potyvirus polyprotein, miraophiovirus RdRp, yuevirus L, and partitivirus RdRp were aligned with representative members of the *Potyviridae*, *Aspiviridae*, *Yueviridiae*, and *Partitiviridae* families, respectively, using the MAFFT [30] aligner with default settings. The best-fit model was determined using ModelTest-NG v0.1.7 [31] for amino acid sequences. The maximum likelihood trees were generated using RaxML-NG v2.0.0 [32] with 1000 bootstrap replicates.

### 2.9. Evaluating Library Contamination

To evaluate whether the libraries were contaminated with other non-viral and non-plant sequences, we utilized kraken2 [33] to assign reads to different OTUs. Briefly, the trimmed reads were used as input files and blasted against a standard kraken2 database with a confidence score of 0.4. Sequence abundance was calculated by counting the number of reads matching the reference sequence OTUs.

## 3. Results

### 3.1. Plant Symptoms

**Figure 1:**
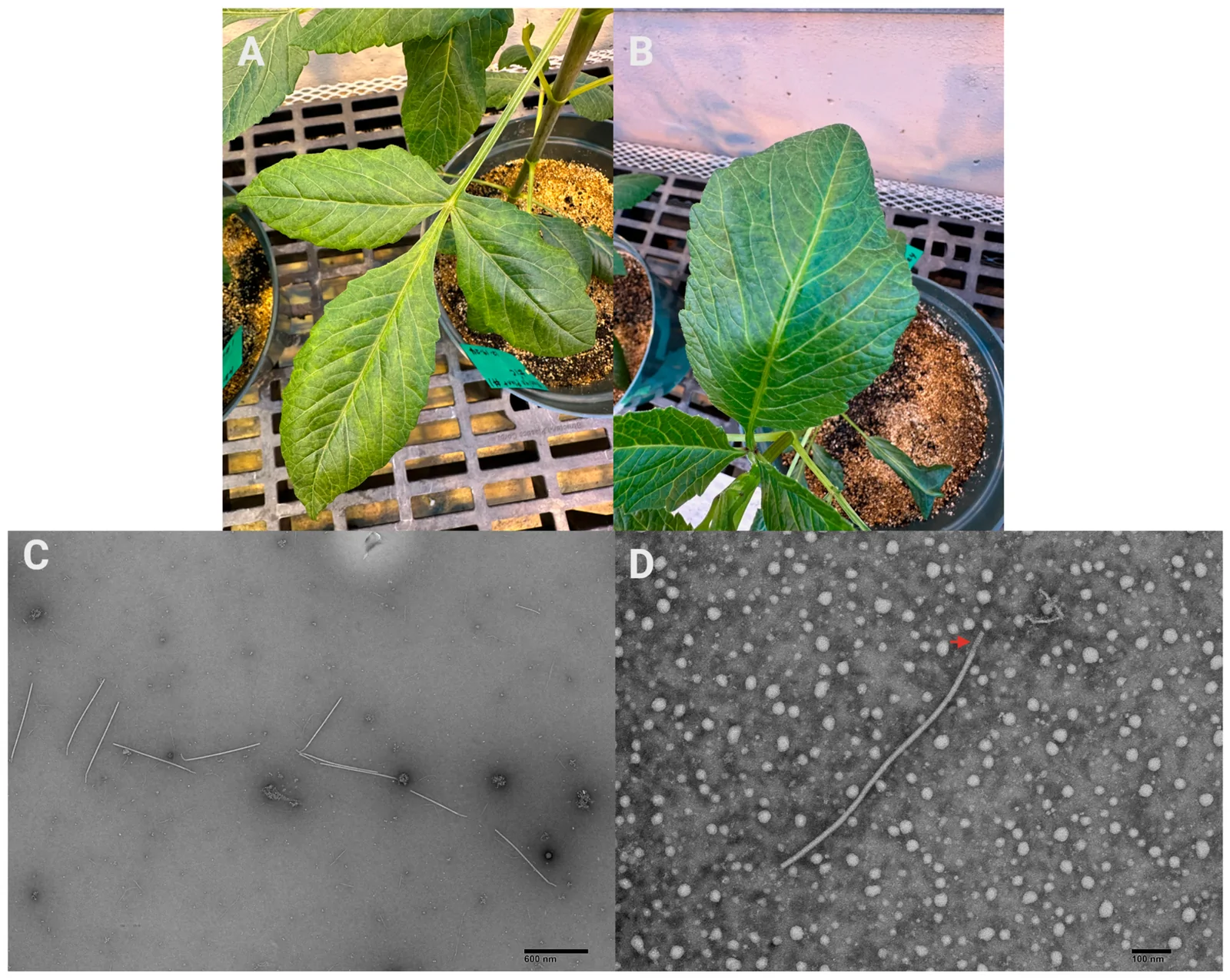
Symptomatic tubers planted in the greenhouse showing mosaic and mottling symptoms. (**A**,**B**) Plants at 10 weeks post-germination. (**C**) Virions shown in transmission electron micrograph of symptomatic Dahlia plants. The image displays the characteristic long, flexuous, filamentous virions of potyvirus. (**D**) The tail of the flexuous virion is indicated by the red arrow.

In California, Dahlia cv. Crichton Honey showed symptoms of leaf mottling during blooming in July. The new leaf growth was green but had stunted growth, which then turned to a mottled yellow and brown. The chlorotic plants completely died back by October. Symptoms on Marilyn plants showed mottling and yellowing. In the greenhouse, plants grown from Marilyn tubers showed mild mottle starting at the third true leaf stage, six weeks after germination (Figure 1A–C). At eight weeks, strong mottling was observed. At twelve weeks, mottle and chlorotic spots were visible throughout the plants. There were no visible differences between the germinated plants that originated from either mother plant, 24-1 or 24-2. The plant flowers did not show any variegation. The plants started to decline during the summer.

### 3.2. HTS Data Summary

A total of 44 M, 17 M, and 24 M raw reads were obtained from samples 22-1, 24-1, and 24-2. Overall, all three samples had an abundant number of viral contigs with hits to variable virus families and genera, such as partitivirus, miraophiovirus, ilarvirus, caulimovirus, and hostuviroid (Table 2). Sample 22-1 had six hits to viral taxonomic entities: potyvirus, yuevirus, miraophiovirus, ilarvirus, caulimovirus and hostuviroid. Sample 22-1 had fragmented contigs with four contigs that gave hits against the Lettuce ring necrosis virus RNA 1. Sample 24-1 had hits to seven virus taxonomic entities: potyvirus, yuevirus, partitivirus, miraophiovirus, ilarvirus, caulimovirus and hostuviroid. Sample 24-1 also had a break in the genome, which yielded two contigs upon re-mapping the reads; the break was resolved by mapping against the contig segment from 24-2. Sample 24-2 had hits to only five virus taxonomic entities: potyvirus, yuevirus, ilarvirus, caulimovirus, and hostuviroid. RT-PCR using specific primers (Table 1) against the viral contigs in the various samples, along with Sanger sequencing, was used to confirm the presence of the viruses.

### 3.3. Characterization of a Novel Potyvirus

**Figure 2:**
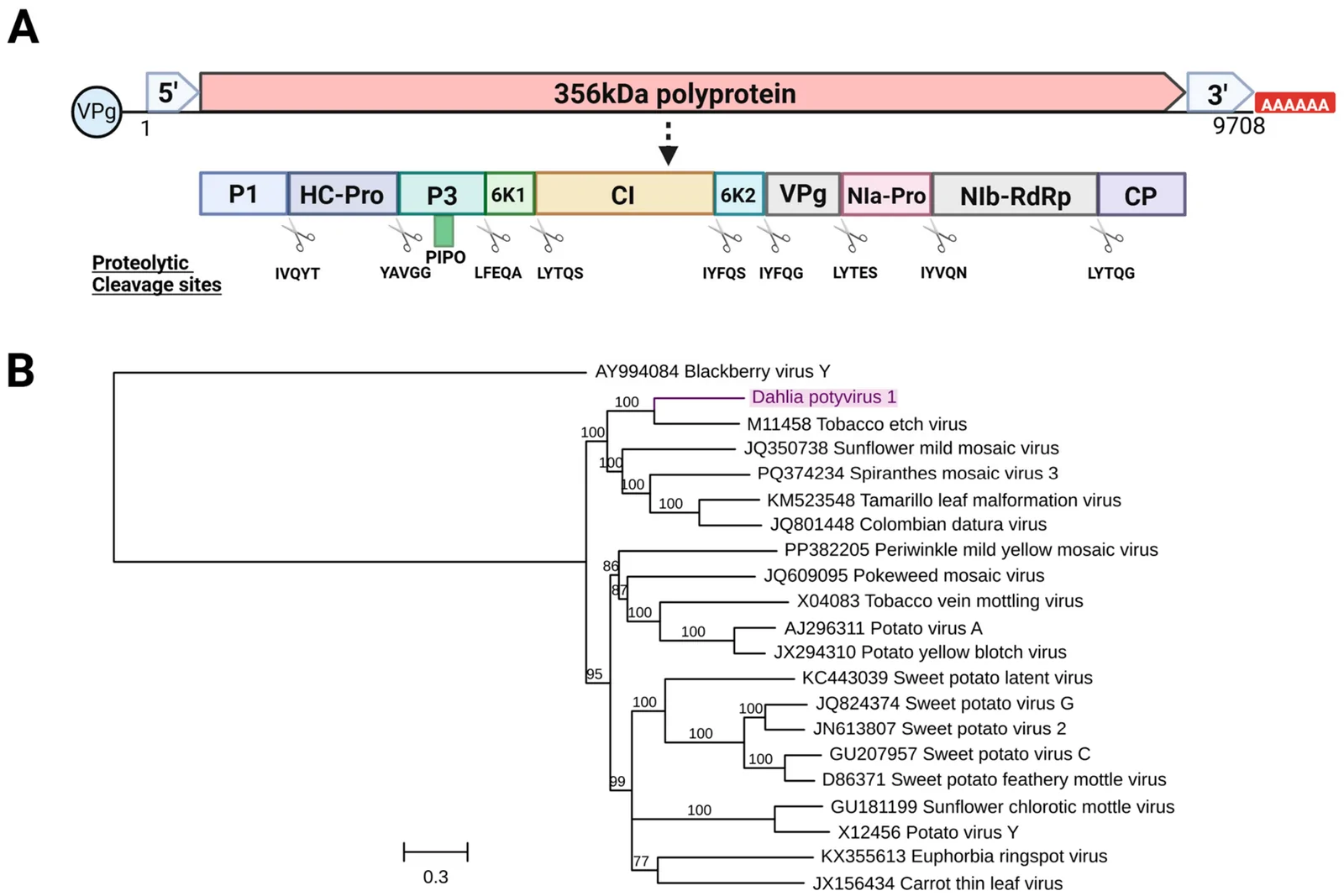
(**A**) Dahlia potyvirus 1 (DaPoV1) schematic representation. The virus encodes a single polyprotein that is self-cleaved into several proteins. Cleavage sites and sequences are indicated between the predicted proteins. The virus has a 5′ terminal VPg (viral protein genome-linked) and a 3′ poly(A) tail with a single large open reading frame (ORF) that encodes a polyprotein processed into ten mature proteins (P1, HC-Pro, P3, 6K1, CI, 6K2, VPg, NIa-Pro, NIb, and CP). The P3-PIPO protein is expressed via a ribosomal frameshift in the P3 region. (**B**) Maximum likelihood tree of the complete amino acid sequence of the polyprotein with other potyviruses. The tree was rooted with the brambyvirus blackberry virus Y, and support values are indicated on branches. Branches with less than 70% bootstrap support were collapsed.

Flexuous rod-shaped virions were observed from leaf dip preparation using TEM. TEM from a partially purified preparation showed both flexuous rod-shaped virions with an average length of 770 nm and width of 14 nm (n = 31). The majority of these virion particles also have a 40 nm long protruding tip that has a more flexuous appearance (Figure 1D,E). Icosahedral-shaped virions of approximately 50 nm in diameter were also observed. A 9.7 kb contig with 63% homology to tobacco etch virus (TEV) was obtained in samples 22-1, 24-1, and 24-2, indicating a novel potyvirus, tentatively named Dahlia potyvirus 1 (DaPoV1). The termini sequences were determined, and the complete genome sequence for DaPoV1 was 9706 nt long, excluding the 3′-terminal poly (A) tail (Figure 2A). The 5′ and 3′ untranslated regions (UTRs) are 115 and 189 nt long, respectively. The genome encodes a putative 3133 aa polyprotein (356.3 kDa) with nine proteolytic cleavage sites that produce ten putative protein products, P1 (42 kDa), HC-Pro (52 kDa), P3 (40 kDa), 6K1 (6 kDa), CI (71 kDa), 6K2 (6 kDa), VPg (21 kDa), NIa-Pro (27 kDa), NIb (59 kDa), and CP (30 kDa), similar to that of other potyviruses (Figure 2A). A +2 ribosomal frameshift at conserved sequence GAAAAAA was present within the P3 domain, which produces the P3N-PIPO, a 79 aa (9 kDa) protein. Several potyvirus conserved motifs were found across different proteins [34,35]. P1 has two motifs, GSSG and FIVRG. HC-Pro has seven motifs: CX_8_CX_18_CX_2_C (zinc finger binding), KITC (aphid transmission), FRNKX_12_CDNQLD (symptomology), IGN (virus movement), CCCVT (long-distance movement), PTK (aphid transmission), and GYCY (cysteine proteinases). The P3 has one motif, EPFX_7_SPX_4_AX_2_NXGX_2_EX_5_W, with putative protease activity. The CI has seven motifs; GAVGSGKST has an NTP-binding function, and the remaining motifs have potential helicase activity: VLMLEPTRPL, DEXH, KVSATPP, LVYV, VATNIIENGVTI, and GERIQRLGRVGR. NIa-Pro has a single motif, HX_34_DX_67_GXCGX_14_H, that contains the catalytic triad of the cysteine protease. NIb-RdRp has six motifs: SLKAEL (RNA polymerase activity), FTAAPID, CVDDFN, AMVESWG, GNNSGQPSTVVDNTLMV, and GDD. The latter two motifs are involved in RNA-dependent polymerase activity. The aphid transmission motif, DAG, was absent, but an alternate motif, NAG, was present in the CP. The remaining six motifs, MVWCIENGTSP, WTMMDGDE, PYMPRYG, AFDF, QMKAAA, and EDTERH, in the CP are of unknown function. The full-length DaPoV1 genome sequence was deposited in GenBank under accession no. PZ668309.

The intragenetic diversity between all three isolates of DaPoV1 showed limited diversity at 99.8–99.9% nt identity. The polyprotein in isolate 24-1 had only five amino acid differences compared to 22-1 and 24-2, two in the P1, one in the HC-Pro, and two in the NIB-RdRp proteins. DaPoV1 shows the highest identity to TEV at 60.1% aa. Phylogenetic analysis placed DaPoV1 within the potyvirus family and shared the highest similarity with TEV at the amino acid level (Figure 2B).

### 3.4. Characterization of a Novel Ophiovirus

**Figure 3:**
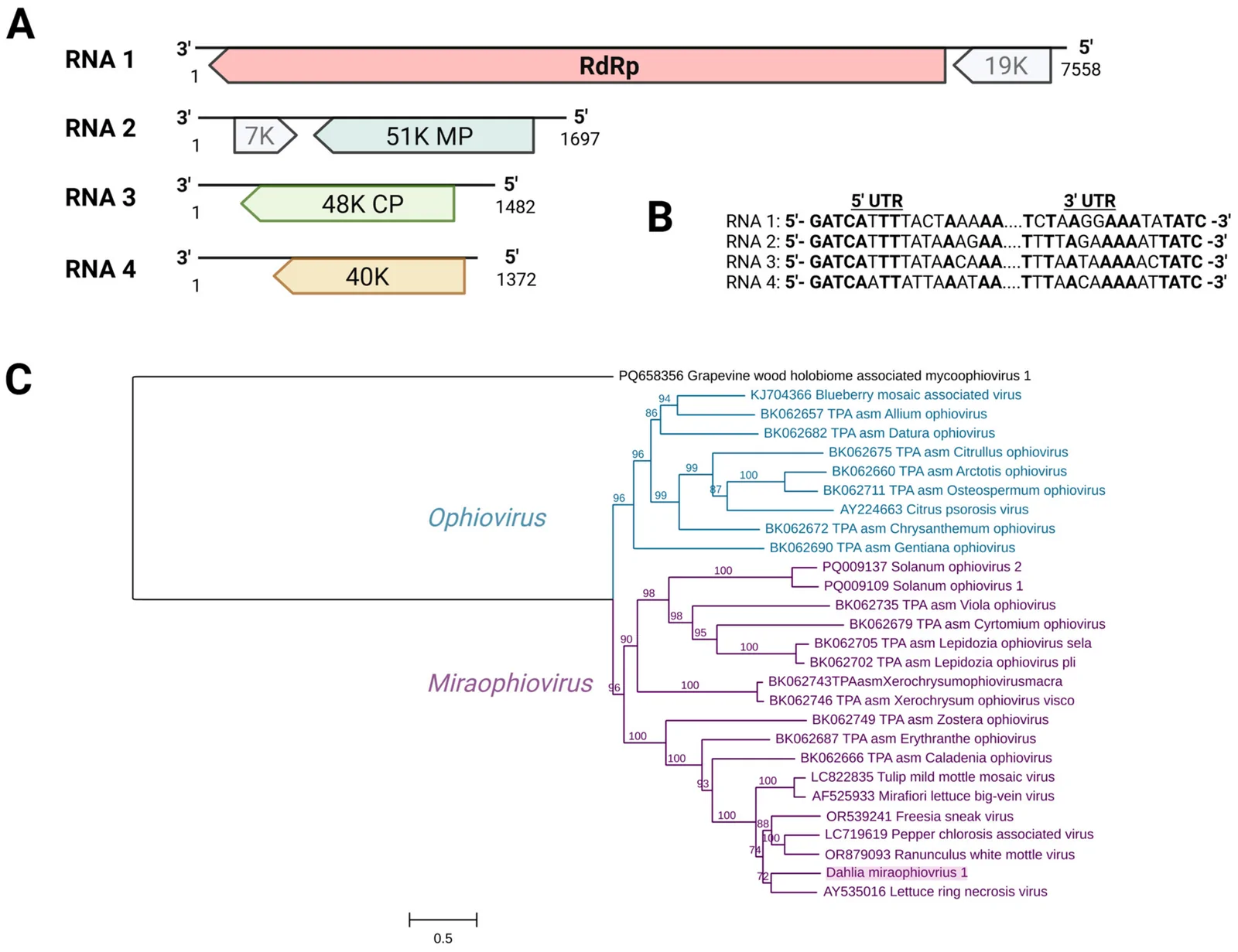
(**A**) Dahlia miraophiovirus 1 (DaMV1) schematic representation of the four RNA segments. RNA1 encodes the RNA-dependent RNA polymerase, (RdRp); RNA2 encodes the movement protein (MP); RNA3 encodes the capsid protein (CP); and RNA4 encodes a hypothetical protein. (**B**) The terminal sequence of the four RNA segments showing high nucleotide homology. Similar bases are highlighted in bold. (**C**) Maximum likelihood tree of the RdRp amino acid sequence with other members of the *Aspiviridae* family. Grapevine wood holobiome-associated mycoophiovirus 1 was used to root the tree, and support values are indicated on branches. Branches with less than 70% bootstrap support were collapsed.

Four contigs with 38 to 62% identity to lettuce ring necrosis virus and mirafiori lettuce big-vein virus were obtained in samples 22-1 and 24-1, indicative of a novel miraophiovirus, tentatively named Dahlia miraophiovirus 1 (DaMV1). RT-PCR assays targeting four RNA segments were performed, and Sanger sequences matched the corresponding contigs. The termini sequences were determined, and the complete genome sequences for DaMV1 RNA 1, 2, 3, and 4 were 7558 nt, 1697 nt, 1482 nt, and 1372 nt long, respectively (Figure 3A). RNA 1 encodes two open reading frames (ORFs) on the viral complementary (vc) RNA strand: the RdRp protein (263 kDa) and a smaller protein (19 kDa) of unknown function, a feature characteristic of the family *Aspiviridae*. RNA 2 encodes two ORFs on the vcRNA, the movement protein (51 kDa) and a smaller protein (7 kDa) of unknown function. RNA 3 encodes only one ORF on the vcRNA, the coat protein (48 kDa). RNA 4 encodes a single ORF on the vcRNA, a protein (40 kDa) of unknown function. RNA 4 does not have a second ORF using a +1 ribosomal frameshift. Analysis of the 5′ UTR and 3′UTR showed very high homology and almost identical nucleotide stretches across the four RNA segments (Figure 3B). The complete genome sequences of DaMV1 for RNA 1, RNA 2, RNA 3, and RNA 4 were deposited in GenBank under accession no. PZ668305, PZ668306, PZ668307, and PZ668308, respectively.

The intragenetic diversity of DaMV1 in 22-1 and 24-1 showed 99.2%, 99.5%, 99.2%, and 99% nt identity for RNAs 1 through 4, respectively. In RNA 1, the RdRp protein and 19K protein showed 99.5% and 98.2% aa identity, respectively. In RNA 2, the 51K MP and 7K proteins showed 99.3% and 100% aa identity, respectively. In RNA 3, the 48K CP showed 99.8% aa identity. In RNA 4, the 40K and 6K proteins showed 98.2% and 98.1% aa identity, respectively. Phylogenetic analysis based on the RdRp and CP amino acid sequences placed DaMV1 within the tentative miraophiovirus clade and nested within the lettuce ring necrosis virus node within the *Aspiviridae* family (Figure 3C).

### 3.5. Characterization of a Novel Partitivirus

**Figure 4:**
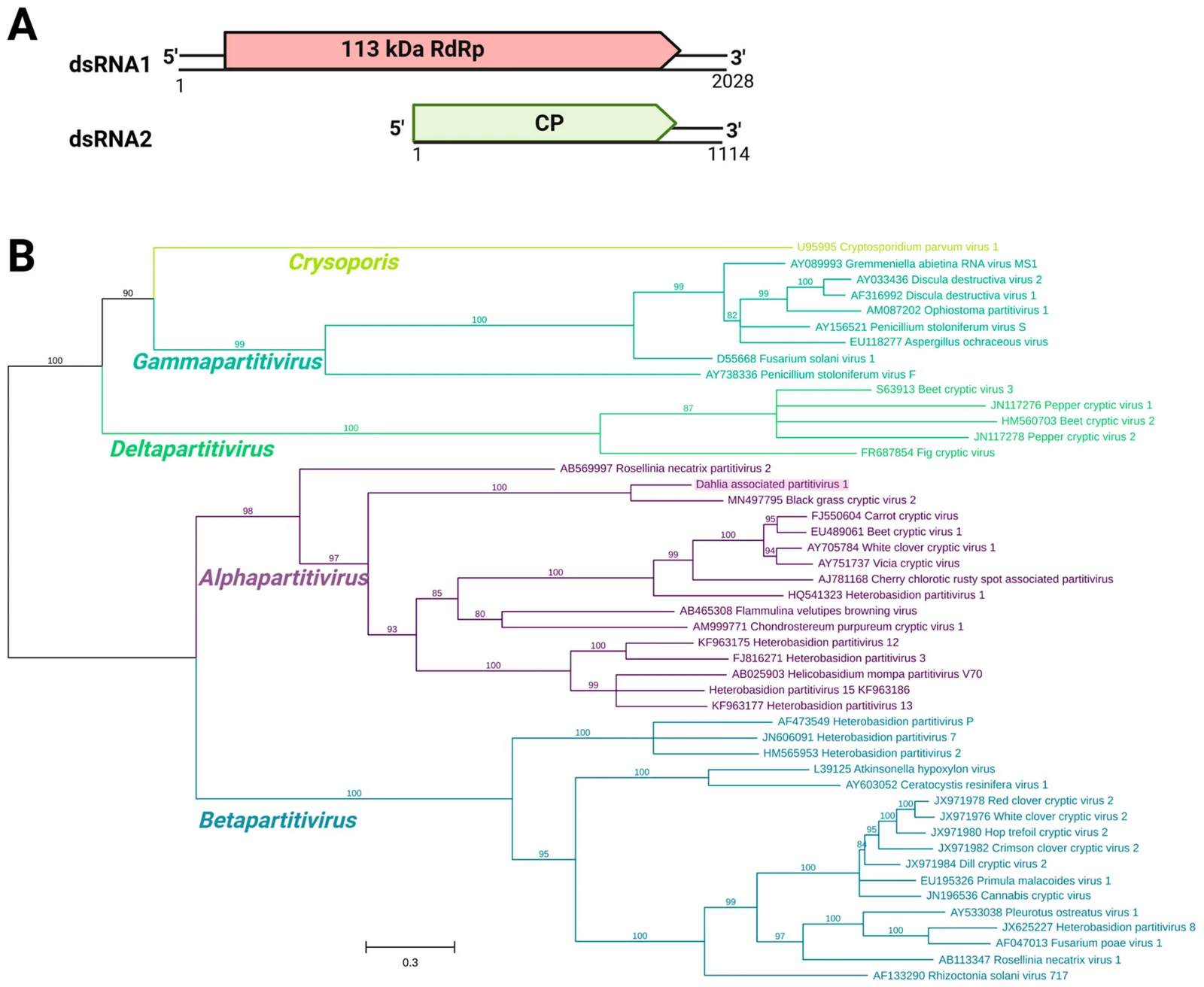
(**A**) Dahlia partitivirus 1 (DaPaV1) schematic representation of the bi-segmented, double-stranded RNA (dsRNA) genome. dsRNA1 contains a single ORF encoding the RNA-dependent RNA polymerase (RdRp), and dsRNA2 encodes the coat protein (CP). Both segments typically feature conserved terminal sequences. (**B**) Maximum likelihood tree of the DaPaV1 RdRp amino acid sequence with other members from the genera *Alphapartitivirus*, *Betapartitivirus*, *Gammapartitivirus*, *Deltapartitivirus*, and *Cryspovirus* in the *Partitiviridae* family. The tree was rooted at the midpoint, and support values are indicated on branches. Branches with less than 70% bootstrap support were collapsed.

Two contigs with 67% and 48% aa homology to black grass cryptic virus 2 RNA 1 and 2, respectively, were obtained in 24-1 only. The number of reads assigned to RNA 2 was very low, indicating low titer in the sample. The complete viral sequence of RNA 1 was determined to be 2028 nt long, and the virus was tentatively named Dahlia-associated partitivirus 1 (DaPaV1) (Figure 4A). RNA 1 encodes a 113 kDa RdRp protein, which is involved in virus replication. The viral sequence of RNA 2 was only partially determined to be 1114 nt long. RNA 2 encodes a coat protein, which is a typical feature of partitiviruses. Phylogenetic analysis placed DaPaV1 within a basal clade along with black grass cryptic virus 2 within the alphapartitiviruses (Figure 4B). The DaPaV1 full-length RNA 1 and partial sequences of RNA 2 were deposited in GenBank under accession no. PZ668302 and PZ668303, respectively.

### 3.6. Characterization of a Novel Yue-like Virus

**Figure 5:**
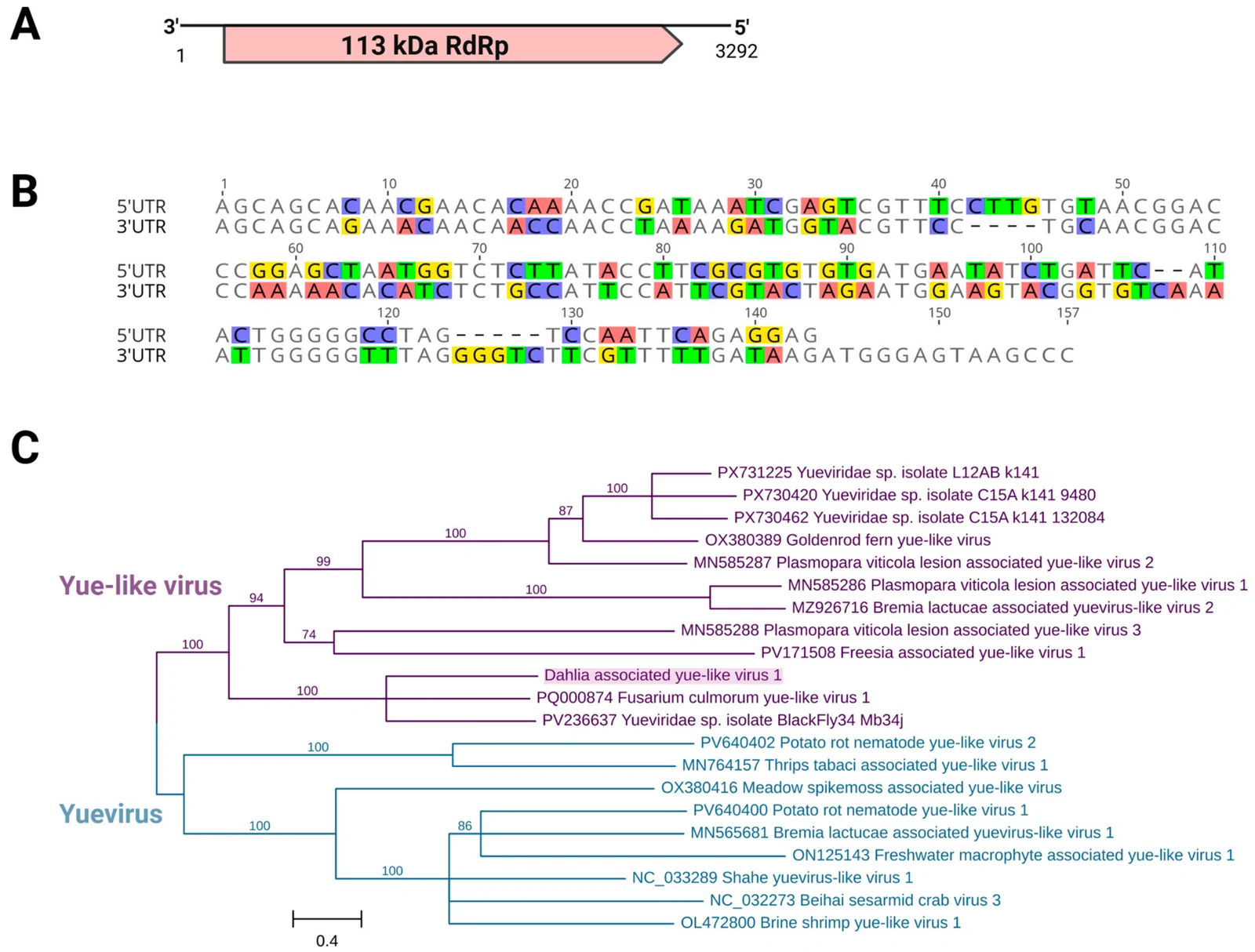
(**A**) Dahlia-associated yue-like virus 1 (DaYV1) schematic representation. The genome contains a single RNA that encodes the L (RdRp) gene. (**B**) The complete 5′ and 3′ untranslated regions (UTRs) of DaYV1 that show complementary termini. (**C**) Maximum likelihood tree of the DaYV1 L amino acid sequence with other members of the Yueviridae family and unclassified yue-like viruses. The tree was rooted at the midpoint, and support values are indicated at branches. Branches with less than 70% bootstrap support were collapsed.

All dahlia samples contained a 3.2 kb contig whose RdRp shares 39.8% amino acid identity with the RdRp of Fusarium culmorum yue-like virus 1, indicative of a novel yuevirus tentatively designated as Dahlia-associated yue-like virus 1 (DaYV1) (Figure 5A). The yuevirus genome consists of a negative-sense RNA with a complete genome sequence of 3292 nt, designated the L segment. The virus is predicted to have a single 3003 nt ORF that encodes a 1000 aa RdRp. The 5′ and 3′ UTRs showed high sequence complementarity with identical eight-nucleotide sequences at each terminus (Figure 5B). We also attempted to identify a second RNA segment, using two approaches: first, screening the assembled contigs with terminal motifs retrieved from the L segment, and by relaxing the blastx identity algorithm against divergent Yuevirus nucleocapsids; neither approach yielded any second segments. The sequences from the three plants showed 100% pairwise nucleotide identity. Phylogenetic analysis placed DaYV1 in a distinct clade with other yue-like viruses that have a similar genome organization and L segment length compared to the yueviruses (Figure 5C). The full-length DaYV1 genome sequence was deposited in GenBank under accession no. PZ668304.

### 3.7. Dahlia-Associated Virome

Several previously characterized viruses were recovered in the various Dahlia samples. All three samples contained DCMV with high nucleotide identity (>90%) to DCMV and not DMV-D10, the endogenous form of DCMV. Two samples, 22-1 and 24-2, had three TSV contigs, representative of all three RNA segments, with >99% identity across the three TSV segments. All three samples contained Dahlia latent viroid (DLVd) with 100% identity to the original reference strain reported by Verhoeven et al. [29].

### 3.8. Transmission Studies

Transmission studies using two sources of inoculum on nine indicators from the *Solanaceae*, one *Amaranthaceae*, and *Cucurbitaceae* plant families did not show any symptom development over a period of one month. Furthermore, RT-PCR testing of non-inoculated leaves at 14 and 28 dpi for all inoculated indicators did not show any successful systemic infection.

### 3.9. Nonviral Targets in Libraries

To evaluate the extent of contamination from other organisms, such as bacteria, fungi, and insects, we utilized kraken2 for the creation of a profile of OTUs. Overall, we found very low contamination of fungal and bacterial reads in the Dahlia dataset. Only 11 reads were assigned to fungal OTUs in both 24-1 and 24-2. Bacterial reads were somewhat higher at 411 and 421 reads in 24-1 and 24-2, respectively. No reads were assigned to Stramenopiles or insects.

## 4. Discussion

Dahlia is an important ornamental in the flower industry and is susceptible to a multitude of viruses. DCMV, TSV, and TSWV have been known for many decades as important viruses in Dahlia. However, recently, symptoms of mosaic, mottling, yellowing, and dieback were observed on Dahlia that are not very typical of these known viruses. To explore the virome of Dahlia, several diagnostic tools, such as TEM, PCR, and HTS, were used. HTS showed that several novel viruses infect Dahlia; however, a strong correlation of the viruses with the observed symptoms is difficult to ascertain due to mixed infections. Whether these viruses cause synergism is unknown and requires further elucidation using infectious clones. Two of these viruses were novel viruses, belonging to the potyvirus and miraophiovirus, both known to cause disease in other hosts. Previously, potyviruses were reported in Dahlia, such as potato virus Y (PVY), bean yellow mosaic virus, and turnip mosaic virus, as an NCBI GenBank accession no. KU512770 [11,36]. In this study, we report the fourth potyvirus and first miraophiovirus in Dahlia. Two other varieties of dahlia plants, Normandy Deegee and Lover Boy, from a nursery were also tested and found to be positive for DaPoV1, indicating that the virus might be more widespread in Dahlia than expected.

DaPoV1 virion shape is intriguing since the terminal end of the virion has a protruding tip that appears to be an incorporation of the HC-Pro and VPg proteins as reported previously for PVY [37]. Meanwhile, only about 10% of PVY virions showed the protruding tips, and most of the DaPoV1 virions showed the protruding tip; whether this is a coincidence of the infection cycle as suggested by Torrance et al. [37] or a characteristic of DaPoV1 is yet to be determined. The majority of the potyvirus motifs were present in DaPoV1 [35]. Interestingly, the DAG motif responsible for aphid transmission was replaced by the NAG motif in the CP of DaPoV1. This suggests that DaPoV1 has lost its aphid transmissibility, as experimentally demonstrated in TEV, plum pox virus, and peanut stripe virus reported in genomes of several other potyviruses [35,38,39,40]. Lopez-Moya et al. [39] showed that mutating the valine upstream of DAG reduces TEV transmission but does not abolish it. The presence of a valine residue immediately upstream of the NAG motif (forming VNAG) may partially preserve structural features associated with aphid transmission. While the aphid transmission motifs, KITC and PTK, are present in the DaPoV1 HC-Pro, interaction with the CP might be weakened with NAG. Therefore, the substitution of the canonical V-DAG motif with V-NAG may either result in a loss or reduction in aphid transmissibility. This could affect virus adaptation to an alternate transmission strategy, likely through vegetative propagation, which may also contribute to the observed narrow host range of DaPoV1.

Ophioviruses in the family *Aspiviridae* are filamentous single-stranded negative-sense RNA viruses that possess three to four segments [41]. Ophioviruses are transmitted by soil-transmitted fungi and have a wide host range ranging from monocots to dicots, trees to vegetables and ornamentals [42]. In this study, we report the first miraophiovirus from Dahlia, expanding the host range of this virus group. Interestingly, DaMV1 clustered with the mirafiori-like clade within the *Aspiviridae*, indicating it belongs to the putative new genus *Miraophiovirus*. The latter is a tentative suggestion based on RdRp and coat protein phylogeny and serological distinction of the mirafiori-like viruses and citrus psorosis viruses [41]. Furthermore, botanically, *Dahlia* and lettuce both belong to the *Asteraceae* family. While we have not tested for the miraophiovirus in our indicator testing, the lack of infection in solanaceous hosts might indicate that these viruses might infect lettuce as an alternative host.

Notably, DaYV1 constitutes the first yue-like virus identified in Dahlia, indicating that yue-like viruses may be more broadly distributed in plants than previously recognized. The *Yueviridae* family includes one genus, *Yuevirus*, that has only two recognized species reported in crustaceans [43]. Further, the Yuevirus are bi-segmented, with a second RNA that encodes the nucleocapsid gene. Recently, several other yue-like viral sequences have been reported in various other non-crustacean hosts, such as plants, insects, and stremenopiles [43]. In plants, yue-like viruses have been found in goldback ferns [43], freesia [44], and spikemoss [45]. Interestingly, these yue-like viruses have only one segment with a smaller replicase protein than the true yueviruses, indicating drastic divergence at the genetic level. Marra et al. [44] noted that plant and stramenopile yue-like viruses cluster in a distinct clade from the true yueviruses, which we have also observed in this study. The mode of transmission and their presence in plants remain unknown. Based on the read assignment results, it is unlikely that fungi or stramenopiles are vectors of yue-like viruses, as their presence in our sample was negligible or absent, respectively. However, that does not preclude prior infection and subsequent persistence of these viruses through vegetative propagation, such as in Dahlia, which is commonly propagated through tubers. Further, whether DaYV1 contributes to disease and symptom development or if this is a latent virus remains to be investigated.

DaPaV1 consisted of two RNA segments as expected for alphaparitiviruses; however, the second segment was partially complete, possibly due to the low virus titer in the plant. Our detection of DaPaV1 expands the known host range of alphapartitiviruses and underscores the growing evidence that partitiviruses may be widespread across diverse plant taxa, including ornamentals such as Dahlia. Although DaPaV1 appeared only in a single sample and at low read abundance, its recovery from plants regenerated from dormant tubers demonstrates that the virus is capable of systemic movement and long-term persistence within Dahlia. This pattern is consistent with many partitiviruses, which are frequently latent, vertically transmitted, and maintained at low titers within their hosts [46,47]. The detection of DaPaV1 in Dahlia therefore raises important questions regarding whether partitiviruses in ornamentals contribute to subtle phenotypic effects, interact synergistically with co-infecting RNA viruses, or remain entirely asymptomatic.

Ornamentals, while highly important for their appearance, pose a threat to commercially cultivated crops due to virus spillovers [48]. Therefore, the surveillance and characterization of viruses in ornamentals remain important to foresee potential emerging viruses. In this study, the potyvirus, yuevirus, ilarvirus, caulimovirus, and hostuviroid were found to be consistently present across the dahlia virome. The miraophiovirus and the partitivirus were not present in all three samples. Nevertheless, all of these viruses appear to be replicated in the plant, as they were obtained in germinating plants from dormant tubers, indicating that the virus was able to systemically move. The accurate determination of disease etiology remains constrained in the absence of infectious clones, and the complexity introduced by mixed infections further obscures definitive attribution of symptom severity to individual viral agents. Therefore, a risk assessment of the viruses causing the observed disease should be considered to align priorities for diagnostics and surveillance. Overall, our finding expands the virus repertoire in Dahlia, which should present as a valuable resource, and reveals that symptoms might be a result of a synergistic interaction between the different viruses.

## Figures and Tables

**Table 1:** Primer sequences used for virus detection and validation.

Purpose	Primer	Sequence (5′–3′)	Targeted Segment	Amplicon Size (bp)
DaPoV1 detection	Dah-HP-cdfaF1	CCCACACAAGGGTACGACAA	HC-Pro	531
	Dah-HP-cdfaR1	GGGTAGCTCTGCGTTGTGCA		
DaMV1 detection	ophR1-F	GGACGCTAAGTTCTCTGACA	RNA1	626
	ophR1-R	AGAATTTCTCCCTCCCCTCT		
	ophR2-F	TCTGTTGTGACACCCATGAA	RNA2	439
	ophR2-R	TCTTGATTTTCGTTGTGGCC		
	ophR3-F	GACTTACGGTTCTGGTAGCA	RNA3	628
	ophR3-R	TGAGCCAAGTGAACTCAACT		
	ophR4-F	ACCACTTGCCTTGATAACCA	RNA4	460
	ophR4-R	TGTCTTGACCTGCTCCTATG		
DCMV detection	DCMV_F	GAACACCACAGCAGAGGAAA	CP	757
	DCMV_R	CCTGGTTTGTCTGTCTGCTT		
TSV detection	TSV-F	TCCAGACCATCCATCCAACG	RNA 3 CP	426
	TSV-R	GTCCCATCAGAGACGGCGA		
DaYV1 virus detection	YueDalF	GTTGGTTCCCAGGAGTCCAG	RdRp	591
	YueDalR	GTCGGCTCTTCGGATCTCAG		
DaPaV1 detection	PartDalF	GCGAACCAGAAACGGCATAC	RdRp	554
	PartDalR	GGGTCTCGGTAAAAAGGGGG		
DLVd detection (Verhoeven et al. [29])	DLVd-P1	GGGGCTCCTCAGAGAGTCTC	Viroid RNA	
	DLVd-P2	GGGGCAACTCCGAGAGTGCTG		

**Table 2:** HTS assembly and viral identification summary.

Family, Genus, Virus	22-1			24-1			24-2		
	Contig (s) Size (bp)	Mapped Reads	Hit (aa %)	Contig (s) Size (bp)	Mapped Reads	Hit (aa %)	Contig (s) Size (bp)	Mapped Reads	Hit (aa %)
Potyviridae; Potyvirus; Dahlia potyvirus 1	9788	5,696,802	Tobacco etch virus; NP_062908.1: 63.3%	5892, 3954	354,909; 239,586	Tobacco etch virus; NP_062908.1: 70%, 51%	9751	985,733	Tobacco etch virus; NP_062908.1: 63.3%
Aspiviridae; Miraophiovirus; Dahlia miraophiovirus 1	2467; 2158; 2016; 864	766; 737; 579; 239	Lettuce ring necrosis virus; YP_053236.1: 65.9%	7572	55,621	Lettuce ring necrosis virus; YP_053236.1: 62.2%	-	-	-
	1655		Lettuce ring necrosis virus; YP_053238.1: 53.5%	1755	112,967	Lettuce ring necrosis virus; YP_053238.1: 53.5%	-	-	-
	1423	2439	Lettuce ring necrosis virus; YP_053239.1: 56.2%	1527	255,135	Lettuce ring necrosis virus; YP_053239.1: 56.5	-	-	-
	1372	2017	Mirafiori lettuce big-vein virus; AWJ64336.1: 37.9%	1381	32,019	Mirafiori lettuce big-vein virus; AWJ64336.1: 38.2%	-	-	-
Yueviridae; unclassified genus; Dahlia yuevirus 1	3264	7367	Fusarium culmorum yue-like virus 1; XCO56027.1: 39.7%	3280	15,322	Fusarium culmorum yue-like virus 1; XCO56027.1: 39.7%	3278	6460	Fusarium culmorum yue-like virus 1; XCO56027.1: 39.7%
Partitviridae; Alphapartitivirus; Dahlia-associated partitivirus 1	-	-	-	1907	352	Black grass cryptic virus 2; YP_009130618.1: 67.8%	-	-	-
	-	-	-	311	15	Black grass cryptic virus 2; YP_009130619.1: 48.5%	-	-	-
Caulimoviridae; Caulimovirus; Dahlia mosaic virus	7654	13,769	Dahlia common mosaic virus; YP_006732334.1: 99.6%	8023	423635	Dahlia common mosaic virus: YP_006732334.1: 88.4%	6206	161,786	Dahlia common mosaic virus; YP_006732334.1: 88.4%
Bromoviridae; Ilarvirus; Tobacco streak virus	3455	35,585	Tobacco streak virus RNA 1; NP_620772.1: 99.9%	-	-	-	3672	734,754	Tobacco streak virus RNA 1; NP_620772.1: 99.5%
	2889	27,094	Tobacco streak virus RNA 2; NP_620768.1: 100%	-	-	-	2940	958,188	Tobacco streak virus RNA 2: NP_620768.1: 99.8%
	2194	27,742	Tobacco streak virus RNA 3; NP_620773.1: 100%	-	-	-	2265	1,569,589	Tobacco streak virus RNA 3; NP_620773.1: 97.2%
Pospiviroidae; Hostuviroid; Dahlia latent viroid	397	1166	Dahlia latent viroid; 100%			Dahlia latent viroid; 100%			Dahlia latent viroid; 100%
Raw reads	44,479,821			17,455,857			24,026,943		

## Data Availability

The raw data supporting the conclusions of this article will be made available by the authors upon reasonable request.

## Supplementary Materials

The following supporting information can be downloaded at: https://www.mdpi.com/article/10.3390/v18090947/s1. Table S1. List of primers used for determining the 5′ and 3′ terminal regions.

## References

[1] Mejía-Muñoz J.M., De Luna-García I., Jiménez-Ruiz E.F., Sosa-Montes E., Flores-Espinosa C., Treviño-De Castro G., Reyes-Santiago J. (2020). Research on dahlia, the national flower of Mexico. Acta Hortic..

[2] Ohno S., Onozaki T. (2025). Dahlia (*Dahlia variabilis* L.) Flower Color and Vase Life. Breeding of Ornamental Crops: Bulbous Flowers.

[3] Burnett S.E., Peterson B.J., Oliveira I., Bowers T. (2023). Comparison of Dahlia Cultivars for Cut Flower Production in the Northeastern United States. HortTechnology.

[4] Şekerci A.D., Gülşen O. (2016). Overview of dahlia breeding. Sci. Pap.-Ser. B Hortic..

[5] Pahalawatta V., Druffel K., Pappu H.R. (2007). Seed transmission of Dahlia mosaic virus in Dahlia pinnata. Plant Dis..

[6] Pappu H.R., Wyatt S.D., Druffel K.L. (2005). Dahlia mosaic virus: Molecular detection and distribution in dahlia in the United States. HortScience.

[7] Geering A.D., McTaggart A.R., Teycheney P.Y. (2022). Untangling the taxonomy of dahlia mosaic virus. Arch. Virol..

[8] Gnanasekaran P., Zhai Y., Pappu H.R. (2026). Genome sequence of dahlia mosaic virus revisited: Molecular characterization and genetic diversity of DMV and dahlia common mosaic virus. Arch. Virol..

[9] Pahalawatta V., Druffel K., Pappu H. (2008). A new and distinct species in the genus Caulimovirus exists as an endogenous plant pararetroviral sequence in its host, *Dahlia variabilis*. Virology.

[10] Pappu H.R., Hammett K.R., Druffel K.L. (2008). Dahlia mosaic virus and Tobacco streak virus in Dahlia (*Dahlia variabilis*) in New Zealand. Plant Dis..

[11] Sastry K.S., Mandal B., Hammond J., Scott S.W., Briddon R.W. (2019). *Dahlia* spp. Encyclopedia of Plant Viruses and Viroids.

[12] Wang S., Zhao Z., Dong Z., Zhou T., Hu Z., Fan Q., Zhang Y. (2024). First report of tomato chlorotic dwarf viroid in dahlia in China. Plant Dis..

[13] Boonham N., Kreuze J., Winter S., van der Vlugt R., Bergervoet J., Tomlinson J., Mumford R. (2014). Methods in virus diagnostics: From ELISA to next generation sequencing. Virus Res..

[14] Jones S., Baizan-Edge A., MacFarlane S., Torrance L. (2017). Viral diagnostics in plants using next generation sequencing: Computational analysis in practice. Front. Plant Sci..

[15] Rott M., Xiang Y., Boyes I., Belton M., Saeed H., Kesanakurti P., Hayes S., Lawrence T., Birch C., Bhagwat B. (2017). Application of next generation sequencing for diagnostic testing of tree fruit viruses and viroids. Plant Dis..

[16] Villamor D.E., Ho T., Al Rwahnih M., Martin R.R., Tzanetakis I.E. (2019). High throughput sequencing for plant virus detection and discovery. Phytopathology.

[17] Saldarelli P., Giampetruzzi A., Maree H.J., Al Rwahnih M. (2017). High-throughput sequencing: Advantages beyond virus identification. Grapevine Viruses: Molecular Biology, Diagnostics and Management.

[18] Wang J., Zhou J., Chen M., Li Z., Song S. (2023). Identification and Genome Characterization of a Dahlia Common Mosaic Virus Isolate from China. Genes.

[19] Yamamoto D., Neriya Y., Suzuki T., Nishigawa H., Natsuaki T. (2021). Construction of an infectious dahlia common mosaic virus clone. Arch. Virol..

[20] Ma Y., Marais A., Lefebvre M., Faure C., Candresse T. (2020). Metagenomic analysis of virome cross-talk between cultivated Solanum lycopersicum and wild Solanum nigrum. Virology.

[21] Debat H., Bejerman N. (2025). An Update on RNA Virus Discovery: Current Challenges and Future Perspectives. Viruses.

[22] Mayhew D.E., Flock R.A. (1981). Sorghum stunt mosaic. Plant Dis..

[23] Abrahamian P., Tian T., Posis K., Guo Y., Yu D., Blomquist C.L., Wei G., Adducci B., Vidalakis G., Bodaghi S. (2024). Genetic analysis of the emerging citrus yellow vein clearing virus reveals a divergent virus population in American isolates. Plant Dis..

[24] Diaz-Lara A., Navarro B., Di Serio F., Stevens K., Hwang M.S., Kohl J., Vu S.T., Falk B.W., Golino D., Al Rwahnih M. (2019). Two novel negative-sense RNA viruses infecting grapevine are members of a newly proposed genus within the family Phenuiviridae. Viruses.

[25] Ha C., Coombs S., Revill P.A., Harding R.M., Vu M., Dale J.L. (2008). Design and application of two novel degenerate primer pairs for the detection and complete genomic characterization of potyviruses. Arch. Virol..

[26] Bolger A.M., Lohse M., Usadel B. (2014). Trimmomatic: A flexible trimmer for Illumina sequence data. Bioinformatics.

[27] Bankevich A., Nurk S., Antipov D., Gurevich A.A., Dvorkin M., Kulikov A.S., Lesin V.M., Nikolenko S.I., Pham S., Prjibelski A.D. (2012). SPAdes: A new genome assembly algorithm and its applications to single-cell sequencing. J. Comput. Biol..

[28] Bushnell B. (2014). BBMap: A Fast, Accurate, Splice-Aware Aligner.

[29] Verhoeven J.T., Meekes E.T., Roenhorst J.W., Flores R., Serra P. (2013). Dahlia latent viroid: A recombinant new species of the family Pospiviroidae posing intriguing questions about its origin and classification. J. Gen. Virol..

[30] Katoh K., Standley D.M. (2013). MAFFT multiple sequence alignment software version 7: Improvements in performance and usability. Mol. Biol. Evol..

[31] Darriba D., Posada D., Kozlov A.M., Stamatakis A., Morel B., Flouri T. (2020). ModelTest-NG: A new and scalable tool for the selection of DNA and protein evolutionary models. Mol. Biol. Evol..

[32] Kozlov A.M., Darriba D., Flouri T., Morel B., Stamatakis A. (2019). RAxML-NG: A fast, scalable and user-friendly tool for maximum likelihood phylogenetic inference. Bioinformatics.

[33] Wood D.E., Lu J., Langmead B. (2019). Improved metagenomic analysis with Kraken 2. Genome Biol..

[34] Worrall E.A., Hayward A.C., Fletcher S.J., Mitter N. (2019). Molecular characterization and analysis of conserved potyviral motifs in bean common mosaic virus (BCMV) for RNAi-mediated protection. Arch. Virol..

[35] Nigam D., LaTourrette K., Souza P.F., Garcia-Ruiz H. (2019). Genome-wide variation in potyviruses. Front. Plant Sci..

[36] Salehzadeh M., Afsharifar A., Farashah S.D. (2023). First report of the incidence of potato virus Y in some ornamental plants in Iran. Iran. Agric. Res..

[37] Torrance L., Andreev I.A., Gabrenaite-Verhovskaya R., Cowan G., Mäkinen K., Taliansky M.E. (2006). An unusual structure at one end of potato potyvirus particles. J. Mol. Biol..

[38] Flasinski S., Cassidy B.G. (1998). Potyvirus aphid transmission requires helper component and homologous coat protein for maximal efficiency. Arch. Virol..

[39] Lopez-Moya J.J., Wang R.Y., Pirone T.P. (1999). Context of the coat protein DAG motif affects potyvirus transmissibility by aphids. J. Gen. Virol..

[40] Kamenova I., Lohuis D., Peters D. (2002). Loss of aphid transmissibility of plum pox virus isolates. Biotechnol. Biotechnol. Equip..

[41] García M.L., Bó E.D., Da Graça J.V., Gago-Zachert S., Hammond J., Moreno P., Natsuaki T., Pallás V., Navarro J.A., Reyes C.A. (2017). ICTV virus taxonomy profile: Ophioviridae. J. Gen. Virol..

[42] Debat H., Garcia M.L., Bejerman N. (2023). Expanding the repertoire of the plant-infecting Ophioviruses through metatranscriptomics data. Viruses.

[43] Krupovic M., Wolf Y.I., Koonin E.V., Kuhn J.H. (2023). ICTV Virus Taxonomy Profile: Yueviridae 2023. J. Gen. Virol..

[44] Marra M., Rotunno S., Frascati F., Pierro R., Hammond J., Vaira A.M., Miozzi L. (2026). The analysis of the virome associated with Freesia refracta plants with necrotic disorder sheds new light on the phylogenetic relationships in the Konkoviridae and Yueviridae families. Virol. J..

[45] Mifsud J.C., Gallagher R.V., Holmes E.C., Geoghegan J.L. (2022). Transcriptome mining expands knowledge of RNA viruses across the plant kingdom. J. Virol..

[46] Roossinck M.J. (2010). Lifestyles of plant viruses. Philos. Trans. R. Soc. B Biol. Sci..

[47] Szego A., Ilyés P., Toth E.K., Potvondi L., Lukacs N. (2004). Long term survival of cryptic viruses in aseptically grown in vitro propagated plants. Acta Hortic..

[48] Hammond J., Huang Q., Jordan R., Meekes E., Fox A., Vazquez-Iglesias I., Vaira A.M., Copetta A., Delmiglio C. (2023). International trade and local effects of viral and bacterial diseases in ornamental plants. Annu. Rev. Phytopathol..

