# Supplementary material for: Dahlia Virome Analysis Reveals Multiple Novel Viruses in Mixed Infections
Source: Viruses. 2026 Aug 29;18(9):947. doi: 10.3390/v18090947 (PMC13612073; doi:10.3390/v18090947)
Supplement: Supplementary file 1 [file viruses-18-00947-s001.zip › viruses-4519507-supplementary.pdf]

Table S1. List of primers used for determining the 5' and 3' terminal regions.

Primer name	Primer Sequence	Target	Region
**DalRa3-F1**	GTGTATAGAGAACGGAACATCA	**Potyvirus**	RACE 3'
**DalRa3-F2**	TAGTGGAAAATGCTCAACCAAC	**Potyvirus**	RACE 3'
**DalRa5-R1**	GCTGTGTTATCCATATCAAGAAC	**Potyvirus**	RACE 5'
**DalRa5-R2**	CAACCGCTGCTGTGTCGT	**Potyvirus**	RACE 5'
**DalRa5-R3**	GTTCCGTGCCAAGTGATTCATGT	**Potyvirus**	RACE 5'
**R1ParR5-1**	ACTTGCTCTAATTCTTCTGGG	**Partitivirus**	RNA 1 RACE 5'
**R1ParR5-2**	TGAGTGACGATTCTTTGGTAAT	**Partitivirus**	RNA 1 RACE 5'
**R1ParR3-1**	GGCTATACCCCAAGTCTTG	**Partitivirus**	RNA 1 RACE 3'
**R1ParR3-2**	GACTTCCCTACCCCACAAG	**Partitivirus**	RNA 1 RACE 3'
**R2ParR5-1**	ACTTGCTCTAATTCTTCTGGG	**Partitivirus**	RNA 2 RACE 5'
**R2ParR5-2**	CTTCAGAGCCAGGGATAATA	**Partitivirus**	RNA 2 RACE 5'
**R2ParR3-1**	TTGCGCTCTGTTGGATTCTG	**Partitivirus**	RNA 2 RACE 3'
**R2ParR3-2**	TATGGCTCGTTTTGCGTCC	**Partitivirus**	RNA 2 RACE 3'
**YueR5-1**	TGTCAGTTGCAGTGAAAGGT	**Yue-like virus**	RACE 5'
**YueR5-2**	GTGTAATCTGGGATGTTCGT	**Yue-like virus**	RACE 5'
**YueR3-1**	GTTCCAGAGATCGTCAAGTG	**Yue-like virus**	RACE 3'
**YueR3-2**	GGAAAGAGATATACTAGCGG	**Yue-like virus**	RACE 3'
**Ra3-r1-F1**	TTCCACTGCTGTAGAAGACCTAA	**Ophiovirus**	RNA 1 RACE 3'
**Ra3-r1-F2**	ACCCTCTGACTTCGGAATTGA	**Ophiovirus**	RNA 1 RACE 3'
**Ra5-r1-R1**	GTTTGAGGTGGTATTTTGGGATT	**Ophiovirus**	RNA 1 RACE 5'
**Ra5-r1-R2**	GGAACCGCAGTCTCGATTATT	**Ophiovirus**	RNA 1 RACE 5'
**Ra3-r2-F1**	TCTTATAACAGGCTCGTGGAAG	**Ophiovirus**	RNA 2 RACE 3'
**Ra3-r2-F2**	TTGTTCCATCAACTAGCACTTCT	**Ophiovirus**	RNA 2 RACE 3'
**Ra5-r2-R1**	TCTCTTTGGGAAACAACGGCAT	**Ophiovirus**	RNA 2 RACE 5'
**Ra5-r2-R2**	ACTCCTTTGAACTCAGAAGCCA	**Ophiovirus**	RNA 2 RACE 5'
**Ra3-r3-F1**	GGCTCAGATTTTGAAGCACAGA	**Ophiovirus**	RNA 3 RACE 3'
**Ra3-r3-F2**	CTGCTATCCTCTTCTCACTTTC	**Ophiovirus**	RNA 3 RACE 3'
**Ra5-r3-R1**	TTGGCTGGATCAATGGAGAATG	**Ophiovirus**	RNA 3 RACE 5'
**Ra5-r3-R2**	AGCTAATTGGGCGATGAAGGA	**Ophiovirus**	RNA 3 RACE 5'
**Ra3-r4-F1**	CCAAAAAGGCCAATTGAACAAGA	**Ophiovirus**	RNA 4 RACE 3'
**Ra3-r4-F2**	GACTACCTTCAACTTCTCAGAG	**Ophiovirus**	RNA 4 RACE 3'
**Ra5-r4-R1**	GGATCATTTCGTTTGGTTTGCC	**Ophiovirus**	RNA 4 RACE 5'
**Ra5-r4-R2**	TCATGTTGTACCACTCTAGAATG	**Ophiovirus**	RNA 4 RACE 5'
